# Reversion mutations in germline *BRCA1/2*-mutant tumors reveal a BRCA-mediated phenotype in non-canonical histologies

**DOI:** 10.1038/s41467-022-34109-8

**Published:** 2022-11-23

**Authors:** Yonina R. Murciano-Goroff, Alison M. Schram, Ezra Y. Rosen, Helen Won, Yixiao Gong, Anne Marie Noronha, Yelena Y. Janjigian, Zsofia K. Stadler, Jason C. Chang, Soo-Ryum Yang, Diana Mandelker, Kenneth Offit, Michael F. Berger, Mark T. A. Donoghue, Chaitanya Bandlamudi, Alexander Drilon

**Affiliations:** 1 Department of Medicine, Memorial Sloan Kettering Cancer Center, New York, NY USA; 2 Weill Cornell Medical College, New York, NY USA; 3 Marie-Josée and Henry R. Kravis Center for Molecular Oncology, Memorial Sloan Kettering Cancer Center, New York, NY USA; 4 Human Oncology and Pathogenesis Program, Memorial Sloan Kettering Cancer Center, New York, NY USA; 5 LOXO Oncology at Lilly, Stamford, CT USA; 6 Department of Pathology and Laboratory Medicine, Memorial Sloan Kettering Cancer Center, New York, NY USA

**Keywords:** Cancer genetics, Cancer genetics, Functional clustering, Cellular signalling networks

## Abstract

The association between loss of *BRCA1/2* and a homologous recombination deficiency phenotype is lineage dependent. In BRCA-associated cancers such as breast, ovarian, pancreas and prostate, this phenotype confers sensitivity to PARP inhibitors and platinum-therapies. Somatic reversion mutations restoring BRCA1/2 function mediate resistance, and have exclusively been reported in BRCA-associated tumors. In this study, we analyze matched tumor and normal sequencing from 31,927 patients and identify 846 (2.7%) patients with germline *BRCA1/2* variants across 43 different cancer types, including 11 with somatic reversion mutations. While nine are in BRCA*-*associated tumors, we find two reversion mutations in non-BRCA-associated histologies, namely lung and esophagogastric adenocarcinomas. Both were detected following platinum therapy. Whole exome sequencing confirms the homologous recombination deficiency phenotype of these tumors. While reversion mutations arise in all BRCA-associated cancer types, here we show that reversion mutations arising post-platinum in non-BRCA associated histologies, while rare, may indicate BRCA1/2 mediated tumorigenesis.

## Abstract (web summary)

Mutations in BRCA1/2 are associated with a homologous recombination deficiency phenotype in BRCA-associated cancers. Reversion mutations can restore BRCA1/2 function and result in treatment resistance in these cancer-types. Here, the authors show that, in select cases, reversion mutations in BRCA1/2 can indicate prior BRCA-mediated tumorigenesis in non-canonical histologies.

## Introduction

Both platinum chemotherapy and poly(adenosine diphosphate ribose) polymerase (PARP) inhibitors induce cell death through DNA damage. *BRCA1/2 (BRCA)* mutations, which inhibit the ability to repair such damage, have been shown to be predictive of responsiveness to these treatments^1–5^.

Recent research has revealed that such a platinum and/or PARP responsive phenotype in *BRCA*-mutant tumors is conditioned on tumor lineage. In classically BRCA-associated tumors, such as breast, ovarian, prostate, and pancreas cancers, *BRCA1/2* behave as drivers, exhibiting zygosity-dependence, selection for biallelic inactivation, and potential benefit from PARP inhibition^6^. By contrast, in other non-canonical BRCA cancer histologies, *BRCA* alterations may be incidental findings rather than drivers of oncogenesis, and are less likely to benefit from PARP inhibition^6^.

While aggregate analysis does not show signs of an HRD phenotype dependent on *BRCA* loss of function (LoF) in non-canonical *BRCA-*mutant tumors, it is unknown whether select *BRCA-*mutant tumors with non-canonical histologies exhibit an HRD phenotype. One method that has been used to confirm a *BRCA*-dependent phenotype is the detection of *BRCA*-reversion mutations following the selective pressure of therapies reliant on the DNA damage pathway. In tumors that exhibit a BRCA*-*mediated HRD phenotype, resistance to treatment with platinum-based chemotherapy and/or PARP inhibition may develop through somatic changes in *BRCA1/2*. Secondary reversion point mutations and insertions or deletions that restore the open reading frame of *BRCA1/2* can lead to recovery of BRCA’s ability to successfully repair the DNA damage induced by PARP inhibitors and platinum-based therapies. In turn, such recovery of BRCA’s functionality in repairing genomic breaks can lead to resistance to PARP inhibitors^5,7–10^ as well as to platinum-based therapy^8,11–14^. The development of reversion mutations in response to the selective pressure of platinum and/or PARP inhibitors, therefore, is considered indicative of the dependence of the original tumor on loss of BRCA function.

In this work, we further characterize the BRCA*-*mediated phenotype across different tumor histologies by examining data from a cohort of over 31,927 patients with diverse tumor types who underwent germline genetic testing and matched tumor next generation sequencing. We identify cases in which reversion mutations indicative of a BRCA-mediated phenotype developed following treatment. While most of the reversion mutations in our cohort are in tumor histologies that have traditionally been thought to be driven by *BRCA1/2*, in this study we highlight the development of reversion mutations in select non-canonical histologies and verify the presence of an HRD phenotype with molecular signatures within whole-exome sequencing (WES).

## Results

### Study cohort

A total of 31,927 patients who underwent prospective matched tumor and normal genomic profiling were included in this study **(**Supplementary Data 1). Overall, 9696 patients (30%) had canonical BRCA-associated tumors (breast, ovarian, prostate, and pancreas cancers) with non-small cell lung cancer (*n* = 4474, 14%) and colorectal (*n* = 2891, 9%) comprising the next two most frequent cancer types represented in our cohort.

### BRCA1/2 germline and somatic mutations

**Fig. 1:**
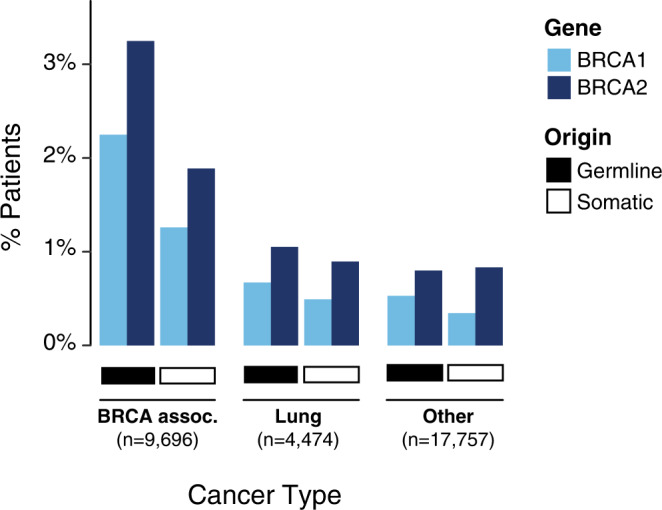
*BRCA1/2* alteration frequency in MSK-IMPACT. Percentage of patients with somatic and germline *BRCA1* and *BRCA2* alterations among patients with *BRCA-*associated cancers (breast, ovarian, pancreas and prostate, *n* = 9696), lung (*n* = 4474), and other cancer types (*n* = 17,757, see Supplementary Data 1 for breakdown of other cancers).

Across all tumor types, 4.5% (*n* = 1422) of patients in our cohort had a germline pathogenic or somatic driver alteration in *BRCA1/2*. The overall prevalence rates of germline pathogenic variants in *BRCA1* and *BRCA2* were 1.1% (*n* = 342) and 1.6% (*n* = 504), respectively. As expected, germline *BRCA1/2* rates were significantly higher in BRCA-associated cancers (overall 5.5%; ovarian 9.6%; pancreatic 5.2%; breast 4.9%; prostate 4.5%) compared to rates in non-*BRCA-*associated histologies (1.4%) (*P* < 0.001, Fishers exact test; Fig. 1). Among the non-*BRCA*-associated lineages with 500 or more patients, the rate of germline *BRCA1/2* prevalence ranged from 2.6% (25/949) in hepatobiliary to 0.5% (3/604) in thyroid, and was 1.7% (77/4474) among patients with non-small cell lung cancer, which was the most common cancer type in our cohort (Supplementary Data 1).

Among the germline wild-type patients, somatic LoF alterations in *BRCA1/2* were identified in 1.8% (*n* = 576) of all patients, with the majority (*n* = 371, 64%) involving *BRCA2*. Similar to germline *BRCA1/2* alterations, somatic *BRCA1/2* alterations were observed at significantly higher frequency in BRCA-associated lineages (overall 3.2%; ovarian 6.5%; prostate 4.6%; breast 2.3%; pancreatic 1.6%) compared to non-BRCA-associated lineages (overall 1.2%) (*P* < 0.001, Fishers exact test). Notably, we observed elevated somatic *BRCA1/2* rates in several non-BRCA-associated lineages such as uterine sarcoma (7.3%), small cell lung cancer (2.6%), and bladder (1.7%) compared to those in *BRCA*-associated lineages. Consistent with prior studies^6,15^, we found these higher rates associated with homozygous deletions at the *BRCA2* locus that often spans the proximal *RB1* that is a common lineage-specific driver in these cancer types.

### Patients with reversion mutations

**Fig. 2:**
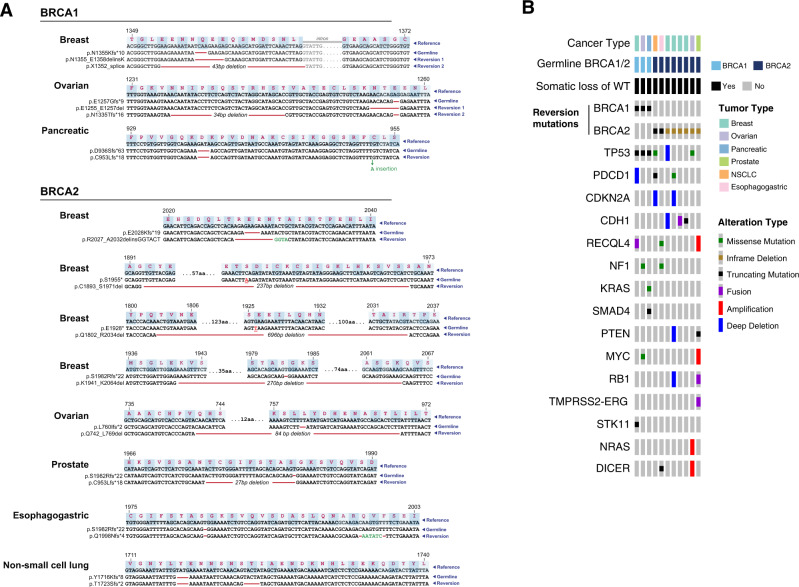
Reversion mutations in germline *BRCA1/2* carriers. **A** The nucleotide and protein coding sequence for the germline and somatic reversion mutations are shown with respect to the reference genome for patients with *BRCA1* (*n* = 3) and *BRCA2* (*n* = 8) somatic reversion mutations. **B** Oncoprint showing clinical characteristics and somatic alterations for all patients with *BRCA1/2* reversion mutations.

We evaluated all patients with germline or somatic truncating mutations in *BRCA1/2* for the presence of a reversion mutation that restored the open reading frame of the mutant allele. In total, eleven patients had germline alterations in *BRCA1/2* (*BRCA1*, *n* = 3; *BRCA2*, *n* = 8), and also were found to have somatic reversion mutations (Fig. 2A). In two patients with germline *BRCA1* variants, one of whom had breast and the other ovarian cancer, we identified two independent reversion mutations indicative of previously observed polyclonal heterogeneity in tumors treated with platinum therapies and/or PARP inhibitors^8^. The 13 reversion mutations we identified comprised 11 deletions affecting up to 696 base pairs, 1 insertion and finally 1 deletion followed by an insertion (Fig. 2A).

Across the eleven patients with reversion mutations, six different cancer types were observed. The three *BRCA1* carriers (one each with breast, ovarian and pancreas cancer) all had tumors with truncating *TP53* mutations which have previously been reported to serve as cooperative mutations in *BRCA1*-deficient germline cancers^16^ (Fig. 2B). Six of the eight germline *BRCA2* carriers with somatic reversion mutations had canonical *BRCA*-associated cancer types, including breast (*n* = 4), ovarian (*n* = 1), and prostate cancers (*n* = 1). The remaining two *BRCA2* germline carriers presented with lung and esophagogastric adenocarcinomas, both tumor types not previously associated with a BRCA-mediated phenotype. Both had family histories of multiple cancers and neither had other canonical disease-specific alterations, such as in *EGFR*, *ALK, KRAS, RET*, *ROS*, *MET*, *ERBB2*, or other common lung or esophagogastric drivers (Fig. 2B). All 11 tumors harbored somatic loss of heterozygosity via copy number loss resulting in loss of the wild-type allele, a hallmark of an HRD phenotype mediated by loss of *BRCA* function^6^.

Detailed clinical data was available for six of the eleven patients including both patients who had non-BRCA associated histology cancers as well as one patient each with ovarian, prostate, pancreas and breast cancers. The six patients comprised three each of *BRCA1* and *BRCA2* carriers. One of the six patients with pancreas cancer, a *BRCA1* carrier, also had a prior history of both breast and ovarian cancer. All patients had some family history of cancer, with a canonical BRCA-associated tumor documented in at least one first-degree relative in all but two patients. In the latter two cases, one patient had a family history of breast cancer in multiple paternal family members and the other had a family history of gastric cancer in a first degree relative.

**Fig. 3:**
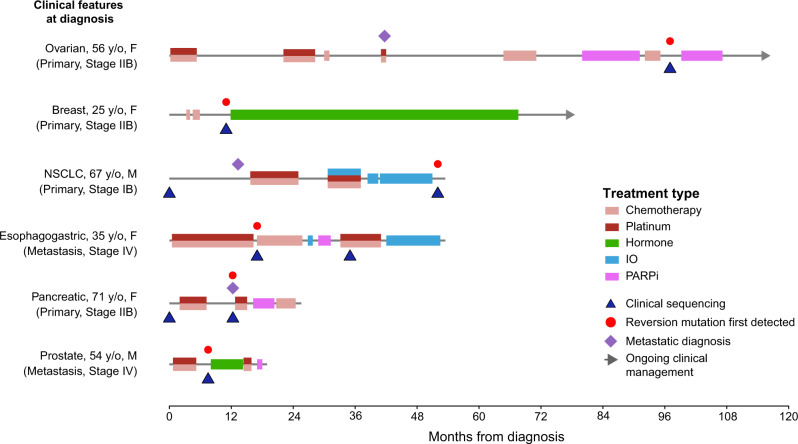
Timeline of treatment for patients whose tumors developed reversion mutations. M male, F female, PARPi poly(adenosine diphosphate ribose) polymerase (PARP) inhibitor, IO immunotherapy.

Five of the six patients received first-line platinum therapy with an overall median time on first-line platinum of 4.8 months (range: 4.1–15.6 months). We identified a reversion mutation in one patient with breast cancer who had not received prior treatment with a PARP inhibitor or platinum agent. The remaining three patients with tumors in canonical BRCA*-*associated histologies were on first-line platinum for a median of 4.5 months (pancreas cancer, 5.1 months; prostate, 4.5 months; ovarian, 4.1 months). The patient with esophagogastric cancer was on first-line platinum therapy for 15.6 months, and the patient with lung cancer was on for 9.2 months (Fig. 3). As neither lung cancer nor esophagogastric cancer are considered canonical BRCA-driven cancers^6^, we report on the cases of these two patients in further detail.

### BRCA2 reversion mutation in a patient with NSCLC

The patient is a male, non-smoker who was diagnosed with a pT2N0M0, stage IB lung adenocarcinoma with acinar and lepidic patterns at age 65 years. He underwent left upper lobectomy, with pathology reportedly TTF-1 positive. Clinical sequencing of the lung tumor biopsy showed a *TP53* (G199V) missense mutation, along with a *BRCA2* Y1716Kfs*8 germline mutation. The patient had an extensive family history, including breast, ovarian, pancreas, colon, and esophagogastric cancers, as well as melanoma in the patient’s relatives. Several of the family members had received their cancer diagnoses at young ages.

Fourteen months after surgery, the patient developed vocal cord paralysis and was found to have a left upper lobe recurrence, as well as a new left adrenal lesion. He was treated with carboplatin and pemetrexed for 6 cycles and achieved a complete response, following which he was continued on maintenance pemetrexed for 7 cycles prior to development of progression of disease in the mediastinum as well as the adrenal glands. He underwent radiation therapy to these sites, and to a subsequently noted metastatic focus in the femur. His adrenal metastasis progressed shortly thereafter, and he was started on nivolumab with paclitaxel for 6 months. Following progression, he enrolled in a trial of an experimental metabolic inhibitor in combination with a checkpoint inhibitor and achieved disease stability. He was then treated with an investigational immunotherapy regimen.

**Fig. 4:**
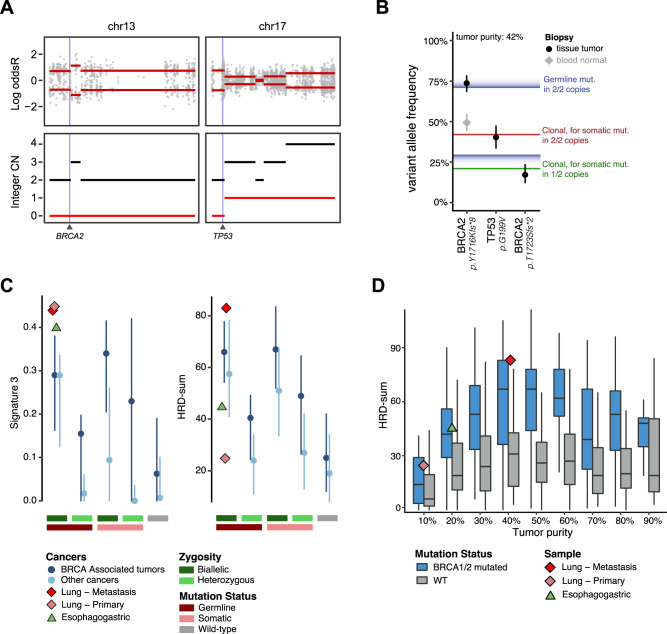
BRCA-mediated phenotypes in lung and esophagogastric cancer cases. **A** In the lung cancer patient, allele-specific copy number profile of the tumor shows a copy-neutral loss of heterozygosity (CNLOH) at both *BRCA2* and *TP53* loci. The top plot shows the log odds ratio for heterozygous SNPs (gray dots). The bottom plot shows integer copy number. The black line represents total copy number, whereas the red line shows minor copy number. **B** Variant allele frequencies in tumor tissue from the patient with lung cancer (black) and matched normal plasma (gray, *BRCA2* germline) are shown for key mutations. The *BRCA2* Y1716Kfs*8 germline variant and somatic *TP53* G199V somatic mutation show allele frequencies in the tumor consistent with complete loss of WT. The reversion mutation is acquired late and is present on one of the two copies of the *BRCA2* allele. Mut = mutation. **C** Left panel shows measures of single nucleotide substitution-based Signature 3 contribution across the previously published 814 exomes from 44 cancer types that are grouped by their *BRCA1/2* mutation and allelic status (Jonsson et al.^6^) along with the two lung (indicated in diamond) and one esophagogastric (in triangle) tumor biopsies. Right panel shows copy-number measures of homologous recombination (HR) deficiency for the same exomes, as measured by HRD-sum, an unweighted sum of large-scale transition (LST), HRD-telomeric allelic imbalance (NtAI) and loss of heterozygosity (HRD-LOH) scores. Dots are median estimates. Error bars show interquartile range (25–75%). **D** HRD-sum scores for all exomes shown in **C** are grouped into deciles of the corresponding tumors’ purity values. The center line of the boxplot is the median, and the lower and the upper hinges represent the first and third quartiles for the HRD-sum scores. The upper and lower whiskers extend up to 1.5× interquartile range above and below the upper and lower hinges, respectively.

He developed a new cutaneous lesion, which was confirmed to be a metastatic focus of lung cancer. Sequencing from the metastatic site continued to show his previously noted *TP53* (G199V) mutation along with the germline *BRCA2* Y1716Kfs*8 mutation. The patient had also developed a new *BRCA2* frameshift deletion in exon 11 (T1723Sfs*2 mutation) that was confirmed to be on the same allele harboring the germline mutation and restored the reading frame, enabling restoration of the functional BRCA2 protein (Fig. 2A). WES of this sample revealed allele-specific copy-neutral loss of heterozygosity at the *BRCA2* and *TP53* loci, with biallelic inactivation of *TP53* (Fig. 4A). The variant allele frequencies of the *BRCA2* germline Y1716Kfs*8 and somatic *TP53* mutations were consistent with complete loss of the wild-type alleles of these genes. The acquired frameshift somatic deletion in *BRCA2* (T1723Sfs*2) was clonal, but only present on one of the two copies of the *BRCA2* allele, both of which harbored the germline mutation (Fig. 4B).

To measure the HRD phenotype in this patient, we performed WES on both the pre-treatment primary and metastatic biopsies. We then calculated single nucleotide substitutions based Signature 3 composition and genomic copy-number based HRD-sum scores^17,18^, measured as unweighted sum of large-scale transitions (LST)^19^, HRD-loss of heterozygosity (HRD-LOH)^20^, and number of telomeric allelic imbalance (NtAI) scores^21^. We then compared these measures of HRD phenotype scores to a subset of the clinical sequencing cohort on whom we previously performed WES. This cohort comprised 814 patients of whom 452 had tumors in canonical BRCA-associated histologies and 66 had lung cancer^6^ (Supplementary Data 2). Overall, 18% (*n* = 148) of the WES cohort harbored germline and 17% (*n* = 139) had somatic *BRCA1/2* mutations. As expected, BRCA-associated histologies with germline mutations in *BRCA1/2* had consistently higher scores across the different measures of HRD phenotype (Fig. 4C).

Similarly, consistent with known hallmarks of a BRCA-mediated phenotype, the metastatic biopsy of the patient with lung cancer had demonstrably higher scores for HRD-sum (83) and Signature 3 composition (0.44) (Fig. 4C). While the pre-treatment primary tumor biopsy also showed high levels of Signature 3 composition (0.45), the HRD-sum score of 25 was notably lower than other samples with germline *BRCA1/2* alterations with concomitant somatic loss of heterozygosity. Given the low tumor purity (14%) of the pretreatment biopsy, we hypothesized that the tumor content dictates sensitivity to detect copy number alterations and is an important determinant of the magnitude of HRD-sum score. To test this hypothesis, we assessed HRD-sum scores relative to tumor purity across the cohort. Consistent with our reasoning, lower purity tumors that were predicted to harbor an HRD phenotype were found to have lower HRD-sum scores (Fig. 4D). Notably, the pretreatment primary tumor biopsy of our patient with lung cancer had an HRD-sum score of 25 that is higher than the median of 14 for *BRCA1/2* mutated tumors at similar purity levels (Fig. 4D), further affirming the HRD phenotype of the pre-treatment lung tumor. Together these observations provide strong evidence for the presence of an HRD phenotype in both the pretreatment primary and metastatic tumor biopsies from the index patient with NSCLC.

### BRCA2 reversion mutation in a patient with gastroesophageal junction adenocarcinoma

The patient was a 35-year-old female who was diagnosed with metastatic adenocarcinoma of the gastroesophageal junction (HER2 negative, MMR proficient, PD-L1 testing not available). The patient’s history was notable for family members with prostate and breast cancer, as well as a case of lung cancer in a non-smoker. Given her young age at presentation and her family history, she was referred for germline genetic testing shortly after diagnosis, which revealed a pathogenic *BRCA2* c.5946delT exon 11 mutation (S1982Rfs*22).

She was started on epirubicin, oxaliplatin, and capecitabine, with excellent radiographic response. After 8 months of treatment, epirubicin was dropped in the setting of decreasing blood counts, and she was continued on oxaliplatin with capecitabine. Thereafter, a PET scan was notable for mild gastric wall uptake, but an upper endoscopy showed an ulcer with no residual malignancy. Overall, she was felt to have achieved a complete response to platinum therapy.

After 15 months on therapy, her PET scan showed possible new sites of disease, including mediastinal, supraclavicular, and level 4 neck nodes, as well as possible inflammatory changes at the hepatic flexure. Clinical sequencing of the gastric mass biopsy showed loss of heterozygosity at the *BRCA2* locus resulting in the loss of the wild-type allele. A reversion mutation in *BRCA*2 at exon 11 (Q1998Nfs*4) that restored the *BRCA2* open reading frame was also identified (Fig. 2A). Additional mutations included a LoF alteration in *DICER1* as well as variants of unknown significance in notable genes such as *FGFR2* (M518L), *NF1* (A1224S) and *RAF1* (S291L). Exome sequencing of this tumor biopsy showed that 40% of all mutations are explained by the Signature 3 mutational process. With a tumor purity of 16%, the HRD sum score of 35 is higher than the corresponding median HRD-sum score of *BRCA1/2* mutated tumors with similar purity levels (Fig. 4C, D). Collectively, these two measures indicate the presence of a robust HRD phenotype in this sample.

Given her progression on platinum-based therapy, the decision was made to switch to irinotecan, initially together with ramucirumab. Following progression, she was enrolled in a clinical trial of immunotherapy, with limited benefit.

She was switched to treatment with the PARP inhibitor olaparib, with paclitaxel later added. Unfortunately, she developed progressive disease associated with significant upper GI bleeding. The PARP inhibitor was discontinued after a little over 5 weeks of treatment.

Radiation therapy was initiated for local control of the bleeding, and she was concomitantly started on carboplatin/paclitaxel, which she was on for approximately 8 months. A further biopsy of the gastric mass while on treatment re-demonstrated the somatic *BRCA2* reversion mutation, in addition to the other previously detected mutations. It also now showed LoF alterations in *ARID1A* (Q766Pfs*51), *CARD11 (R75Q)*, and *KMT2C* (P1962Lfs*8).

Thereafter, she received sixth line chemotherapy and seventh line immunotherapy, as well as additional radiation both systemically and to brain metastases. She unfortunately passed away from her disease 4.3 years after diagnosis. Overall, we report a case of esophagogastric cancer with a germline *BRCA2* alteration who achieved a complete response to platinum. When her tumor later developed platinum resistance, it was found to have a reversion mutation that restored BRCA2 functionality.

## Discussion

Our analysis of genomic data from 31,927 patients with matched germline and tumor sequencing from a variety of cancer histologies reveals *BRCA1/2* reversion mutations across BRCA*-*associated tumor types. Interestingly, mining data from our clinical database also revealed rare cases in which non-canonical histology tumors developed reversion mutations after the selective pressure of platinum-based therapy, suggestive of an initial phenotype mediated by loss of BRCA function.

Prior work from our group has shown that a BRCA-mediated HRD phenotype is lineage specific, with such a phenotype more commonly seen in BRCA*-*associated tumor types^6^. The incidence of an HRD phenotype mediated by BRCA in non-canonical histologies is unclear. By analyzing reversion mutations across a large pan-cancer cohort, we identified two cases of *BRCA* reversion mutations in patients with non-canonical tumor histologies, namely in lung and esophagogastric cancers.

Although rare germline alterations in *BRCA1/2* have been reported in association with squamous cell and other lung cancers^22–27^, evidence for the role of *BRCA1/2* alterations in the pathogenesis of lung cancers is sparse^28^. For example, the rate of loss of heterozygosity among germline carriers of *BRCA1/2* mutations is not significantly higher than that of the background rate measured for benign variants in lung cancer^6^.

Contrasting such aggregate data, both the clinical context of our patient with lung cancer’s reversion mutation developing after platinum-based treatment, and the pathologic context suggest a BRCA LoF-mediated phenotype. The tumor showed no other recognized driver alterations, and the only other significant mutation was biallelic loss of *TP53*. While the latter is a frequent alteration across all cancer histologies^29^, it has also been more specifically associated with an HRD phenotype in patients with *BRCA* mutations and with locus-specific loss of heterozygosity^30^. Mutations in *TP53* were also the most common co-alterations in patients with reversion mutations in our cohort, regardless of histology. In addition to the fact that *TP53* was the only significant co-alteration seen in our patient with lung cancer, comparison of WES with HRD-associated genomic signatures from 814 other tumors of diverse histologies revealed strong concordance with the pattern exhibited by classic BRCA-associated malignancies.

Our analysis of *BRCA1/2* alterations across a large database of patients also revealed a *BRCA* reversion mutation in a patient with an adenocarcinoma of the gastroesophageal junction. Although neither esophageal nor gastric cancer are considered canonical BRCA*-*driven tumors, there have been reports of both tumor types in patients with *BRCA* mutations^31–33^, including in patients with diffuse gastric carcinomas^34^ and squamous cell carcinoma of the esophagus^35,36^. In one study comparing cancer incidence in 490 families with *BRCA1/2* mutations to local population-based cancer estimates from North West England, an increased relative risk of both esophageal and gastric cancer was identified in *BRCA* carriers^37^. However, the question of whether BRCA is truly a pathogenic driver in these cancers remains, and reversion mutations have not been reported. Our patient with adenocarcinoma of the gastroesophageal junction had a deep and prolonged response to platinum-based therapy, with detection of a reversion mutation at the time of progression. This history is suggestive of an initial BRCA-mediated HRD phenotype, with the reversion mutation restoring the tumor’s DNA repair functions and hence driving resistance. Notably, the patient had rapid clinical deterioration when a PARP inhibitor was trialed after the development of the reversion mutation, potentially due to the role of the reversion in restoring wildtype BRCA functionality. Whole exome analysis again further affirmed an HRD phenotype.

Although our analysis shows that some non-canonical histology tumors may have a BRCA-mediated HRD phenotype, it also confirms that such instances are rare. In our cohort of over 30,000 patients, the majority of patients with *BRCA* reversion mutations had breast, ovarian, prostate, or pancreas cancers and only two reversion alterations in non-canonical histologies were identified. Prospectively identifying those select patients with non-canonical histology tumors who have an HRD-phenotype will require further assessment of companion diagnostic assays in clinical trials. For example, in canonical histologies, there have been early efforts to correlate responses to PARP and platinum-based therapies with the results of the RAD51 foci and whole-genome based HRDetect assays as markers of homologous recombination activity^38–42^. Whether such assays can be used to prospectively identify those rare patients with non-canonical histology tumors who may derive robust benefit from platinum or PARP inhibitor therapy requires further clinical investigation. In other words, while the emergence of reversion mutations as a mechanism of resistance to platinum-based therapy can retrospectively identify tumors dependent on loss of BRCA function, further research will be needed to prospectively identify which patients with non-canonical histology cancers have BRCA*-*mediated tumors prior to receipt of treatments capitalizing on an HRD phenotype.

Our analysis of reversion mutations in a pan-cancer cohort has important limitations. First, while *BRCA*-reversion mutations following the selective pressure of DNA-damaging agents are suggestive of a BRCA-mediated phenotype, we cannot completely exclude the possibility that reversion mutations in some tumors occur as random events. Indeed, there was one case of a patient in our cohort with breast cancer and a reversion mutation in *BRCA1*, with no documented history of having received either a PARP inhibitor or a platinum-based therapy, the two classes of therapy that have been associated with reversion alterations due to their role in capitalizing on the DNA damage repair pathway. The patient had received prior chemotherapy, including with doxorubicin (an intercalating agent), cyclophosphamide (a DNA cross-linking agent), as well as both docetaxel and paclitaxel (agents impacting microtubular function). Whether the reversion mutation in this patient with breast cancer emerged as a resistance mechanism to these chemotherapies or occurred spontaneously is unknown. Importantly, we did attempt to mitigate the possibility that reversion mutations in the cohort merely reflected broader genomic instability and that resistance to drugs affecting the DNA damage repair pathway could have occurred through other mechanisms by excluding those patients with high TMB from the analysis. In the case of our patients with gastroesophageal and lung cancers, WES biopsies revealed HRD signatures typical of BRCA-associated tumors, including on sequencing of both a primary and metastatic site in the case of the patient with lung cancer. These findings were suggestive of true dependence of the original tumor phenotype on loss of BRCA function and the emergence of the reversion as a mechanism of resistance to platinum-based therapy.

A further limitation of our analysis derives from the fact that our clinical sequencing efforts aim to biopsy and profile tumors at the time of diagnosis or at the time of referral to our Center. Because many patients whose tumors are profiled are treatment naïve, our analysis likely underestimates the prevalence of reversion mutations and may not capture all histologies in which reversions occur.

In conclusion, while most BRCA-driven tumors are breast, ovarian, prostate, or pancreas cancers, analysis of *BRCA* reversion mutations in a large pan-cancer cohort reveals very rare cases of lung and esophagogastric cancers mediated by loss of BRCA function. Reversion mutations occurring after receipt of platinum-therapy may reflect rare cases of a BRCA-mediated phenotype in non-canonical tumor histologies.

## Methods

### Study cohort

The study cohort comprised 34,036 patients who underwent prospective matched tumor and normal sequencing using our FDA-authorized MSK-IMPACT clinical assay (between January 2014 and July 2019) as part of their active clinical care at Memorial Sloan Kettering Cancer Center (MSKCC)^29,43^. All patients were provided informed consent and accrued for sequencing under the Institutional Review Board approved research protocol #12-245. A total of 12,803 patients consented to germline testing to assess known cancer predisposition genes, which allowed matching of genomic data to detailed clinical and pathologic information^44^. For the remaining patients, somatic and limited clinical attributes were anonymized prior to germline variant discovery and downstream integrated somatic and germline analyses. We excluded 2109 patients whose tumors presented with high tumor mutational burden (>20 nonysnonymous mutations per megabase) where somatic *BRCA1/2* mutations could be attributed to aberrant mutational processes. The remaining 31,927 patients encompassing 73 different cancer types were included in this analysis.

### Pathogenic germline and somatic mutations

We performed germline variant calling using a clinical validated pipeline for specimens sequenced using MSK-IMPACT panel in a CLIA-compliant laboratory^44^. We inferred germline pathogenic variants in *BRCA1/2* using a random-forest based binary classifier for pathogenicity that is trained on an expert curated list of pathogenic variants that satisfy ACMG guidelines for clinical interpretation^44,45^. Features for the classifier included known pathogenic variants in ClinVar, population frequencies of variants, variant type (missense, truncating, etc.), type of gene (oncogene vs. tumor suppressor), in silico prediction scores for underlying sequence conservation, protein family annotation and three-dimensional protein structure data. We also performed additional filtering to remove C-terminal variants in the last exon of *BRCA1/2* genes that are predicted to have no effect on the enzymatic domains. Somatic nonsynonymous mutations (substitutions, insertions and deletions), gene-level amplifications and deletions, and fusions were called using our clinical pipeline^29,43^. All somatic alterations in *BRCA1/2* were classified as LoF if they were annotated as “oncogenic” or “likely oncogenic” in the FDA-recognized precision oncology knowledgebase, OncoKB^46^.

### Identification of reversion mutations

Tumors of patients harboring frameshift or nonsense germline variants in *BRCA1/2* were analyzed for somatic insertion and deletions that restore the open reading frame. We first identified 19 germline carriers of *BRCA1/2* truncating mutations with somatic insertions or deletions within 200 amino acids of the germline variant. To determine whether the somatic mutation is on the same allele as the germline variant, we manually reviewed the aligned reads^47^ in these patients to identify paired-reads spanning both germline and somatic alteration sites. At least three paired-reads each spanning both the germline and somatic alterations were required to establish that the somatic mutation was in *cis* with the germline variant. For large somatic deletions that fully encompassed the germline variant (Fig. 2A), such evidence from a single paired-read supporting both the germline variant and the somatic alteration is not obtainable. In such instances, we leveraged the allelic imbalance status, hypothesizing that the presence of a clonal loss of wild-type event will result in only the allele with the germline variant being retained and therefore this allele being the only substrate that could be somatically mutated. The allelic copy number state was determined using FACETS v0.5.14^48^. Subsequently, alleles with both alterations in *cis* were evaluated for restoration of the open reading frame.

### Exome capture and sequencing of lung and esophagogastric tumors

WES was carried out by the Integrated Genomics Operations Core of Memorial Sloan Kettering Cancer Center (New York, NY). In all, 396–500 ng of barcoded library were captured by hybridization using the xGen Exome Research Panel v1.0 (IDT) according to the manufacturer’s protocol. PCR amplification of the post-capture libraries was carried out for 12 cycles. Samples were run on a HiSeq 4000 in a PE100 run, using the HiSeq 3000/4000 SBS Kit (Illumina). Normal and tumor samples were covered to an average of 141× and 198×, respectively. WES was processed and analyzed using the TEMPO pipeline (v1.3, https://ccstempo.netlify.app/). In brief, demultiplexed FASTQ files were aligned to the b37 assembly of the human reference genome from the GATK bundle using BWA mem (v0.7.17). Aligned reads were converted and sorted into BAM files using samtools (v1.9) and marked for PCR duplicates using GATK MarkDuplicates (v3.8-1). Somatic mutations (single-nucleotide variants and small insertions and deletions) were called in tumor–normal pairs using MuTect2 (v4.1.0.0) and Strelka2 (v2.9.10), and structural variants were detected using Delly (v0.8.2) and Manta (v1.5.0). Somatic mutations were filtered as follows^6^. All variants that were annotated as “oncogenic” and “likely oncogenic” using OncoKB^46^ are whitelisted. All non-whitelisted variants were filtered to exclude those: (1) occurring in repetitive or low-complexity regions annotated by ENCODE consortium (https://hgdownload.cse.ucsc.edu/goldenPath/hg19/database/rmsk.txt.gz and https://genome.ucsc.edu/cgi-bin/hgFileUi?db=hg19&g=wgEncodeMapability), (2) found in 10 or more patients in non-cancer patients in gnomAD [ref], (3) that have low variant allele frequency (<5%) and are supported by three or fewer reads in regions with low coverage (<20×).

### Tumor zygosity, mutational signatures, and HRD scores

Measures of homologous recombination repair deficiency such as large-scale transition (LST), HRD-loss of heterozygosity (HRD-LOH) and number of telomeric allelic imbalance (NtAI) scores were calculated using the facets-suite v2.0.6 package (https://github.com/mskcc/facets-suite)^6,49–51^. Allele-specific copy number segmentation calls for all exomes were inferred by running FACETS v0.5.14 algorithm in a two-pass approach. While the first pass (cval = 500) determined the diploid state, the second pass (cval = 100) inferred the copy number states of individual segments which are used to compute the HRD scores. Mutational signatures were inferred from all single-nucleotide mutations using a maximum likelihood-based extraction approach that determines mutational signature proportions for a set of mutation count data under a known set of COSMIC Version 2^52^ signatures^6^ (https://github.com/mskcc/mutation-signatures). Biallelic status of *BRCA1/2* germline and somatic mutations were inferred using allele-specific copy number estimate at each locus, tumor purity and observed variant allele frequency in the tumor^6,45^. Patients with a *BRCA1/2* germline or somatic LoF mutation who also harbor a homozygous deletion, a fusion event or a second somatic mutation are also considered to be biallelic.

### Statistical analysis

Comparisons of rates of *BRCA1/2* mutations in different tumor types were carried out using Fisher’s Exact Test. Statistical analysis was carried out using either R (R Core Team, Vienna, Austria) or Graphpad version 8 (La Jolla, CA, USA). The threshold for statistical significance was *p* < 0.05.

### Reporting summary

Further information on research design is available in the Nature Research Reporting Summary linked to this article.

## Supplementary information

Description of Additional Supplementary Files

Supplementary Data 1

Supplementary table 2

Reporting Summary

## Data Availability

MSK-IMPACT sequencing data are considered protected information and access to raw data is therefore restricted. The whole-exome sequencing data are available in the NCBI dbGaP archive under accession numbers phs001783.v4.p1. Access via the NCI’s dbGAP can be requested by qualified senior and principle investigators overseeing the research. The NCI’s Data Access Committee reviews such requests within 2 weeks and will make data available for up to 12 months. Requests for access may be directed to Michael Berger (bergerm1@mskcc.org). All other data are available in the Supplementary Data or Source data files accompanying this manuscript. Source data are provided with this paper.

## Source data

Source data file
