# Supplementary material for: Reversion mutations in germline BRCA1/2-mutant tumors reveal a BRCA-mediated phenotype in non-canonical histologies
Source: Nat Commun. 2022 Nov 23;13:7182. doi: 10.1038/s41467-022-34109-8 (PMC9684575; doi:10.1038/s41467-022-34109-8)
Supplement: Supplementary file 1 — Description of Additional Supplementary Files [file 41467_2022_34109_MOESM1_ESM.docx]

**Description of Additional Supplementary Files**

File Name: Supplementary Data 1

Description: Demographics of the examined cohort of 31,927 patients with matched tumor and normal genomic profiling.

File Name: Supplementary Data 2

Description: HRD and mutational signature 3 scores for all exomes included in this study.
