# Supplementary material for: Reversion mutations in germline BRCA1/2-mutant tumors reveal a BRCA-mediated phenotype in non-canonical histologies
Source: Nat Commun. 2022 Nov 23;13:7182. doi: 10.1038/s41467-022-34109-8 (PMC9684575; doi:10.1038/s41467-022-34109-8)
Supplement: Supplementary file 4 — Reporting Summary [file 41467_2022_34109_MOESM4_ESM.pdf]

nature portfolio

Corresponding author(s): Alexander Drilon
Chaitanya Bandlamudi

Last updated by author(s): May 18, 2022

Reporting Summary

Nature Portfolio wishes to improve the reproducibility of the work that we publish. This form provides structure for consistency and transparency in reporting. For further information on Nature Portfolio policies, see our Editorial Policies and the Editorial Policy Checklist.

Statistics

For all statistical analyses, confirm that the following items are present in the figure legend, table legend, main text, or Methods section.

n/a	Confirmed	
☐	☒	The exact sample size (n) for each experimental group/condition, given as a discrete number and unit of measurement
☐	☒	A statement on whether measurements were taken from distinct samples or whether the same sample was measured repeatedly
☐	☒	The statistical test(s) used AND whether they are one- or two-sided<br>*Only common tests should be described solely by name; describe more complex techniques in the Methods section.*
☐	☒	A description of all covariates tested
☐	☒	A description of any assumptions or corrections, such as tests of normality and adjustment for multiple comparisons
☐	☒	A full description of the statistical parameters including central tendency (e.g. means) or other basic estimates (e.g. regression coefficient) AND variation (e.g. standard deviation) or associated estimates of uncertainty (e.g. confidence intervals)
☐	☒	For null hypothesis testing, the test statistic (e.g. F, t, r) with confidence intervals, effect sizes, degrees of freedom and P value noted<br>*Give P values as exact values whenever suitable.*
☒	☐	For Bayesian analysis, information on the choice of priors and Markov chain Monte Carlo settings
☒	☐	For hierarchical and complex designs, identification of the appropriate level for tests and full reporting of outcomes
☒	☐	Estimates of effect sizes (e.g. Cohen's d, Pearson's r), indicating how they were calculated

Our web collection on statistics for biologists contains articles on many of the points above.

Software and code

Policy information about availability of computer code

Data collection	No code was used for data collection
Data analysis	R, Graphpad,<br>FACETS v0.5.14 - https://github.com/mskcc/facets<br>OncoKB - www.oncokb.org

For manuscripts utilizing custom algorithms or software that are central to the research but not yet described in published literature, software must be made available to editors and reviewers. We strongly encourage code deposition in a community repository (e.g. GitHub). See the Nature Portfolio guidelines for submitting code & software for further information.

Data

Policy information about availability of data

All manuscripts must include a data availability statement. This statement should provide the following information, where applicable:

- Accession codes, unique identifiers, or web links for publicly available datasets
- A description of any restrictions on data availability
- For clinical datasets or third party data, please ensure that the statement adheres to our policy

The whole-exome sequencing data are pending deposition in the NCBI dbGaP archive under accession numbers phs001783. All other data supporting the findings of this study are available within the article.

Field-specific reporting

Please select the one below that is the best fit for your research. If you are not sure, read the appropriate sections before making your selection.

- [x] Life sciences
- [ ] Behavioural & social sciences
- [ ] Ecological, evolutionary & environmental sciences

For a reference copy of the document with all sections, see nature.com/documents/nr-reporting-summary-flat.pdf

Life sciences study design

All studies must disclose on these points even when the disclosure is negative.

Sample size	The study investigated a retrospective cohort, comprised of 34,036 patients who underwent prospective matched tumor and normal sequencing using our FDA-authorized MSK-IMPACT clinical assay.
Data exclusions	No data were excluded
Replication	Not applicable.
Randomization	Not applicable.
Blinding	Not applicable.

Reporting for specific materials, systems and methods

We require information from authors about some types of materials, experimental systems and methods used in many studies. Here, indicate whether each material, system or method listed is relevant to your study. If you are not sure if a list item applies to your research, read the appropriate section before selecting a response.

Materials & experimental systems

n/a	Involved in the study	
x		Antibodies
x		Eukaryotic cell lines
x		Palaeontology and archaeology
x		Animals and other organisms
	x	Human research participants
	x	Clinical data
x		Dual use research of concern

Methods

n/a	Involved in the study	
x		ChIP-seq
x		Flow cytometry
x		MRI-based neuroimaging

Human research participants

Policy information about studies involving human research participants

Population characteristics	Data on the characteristics of the patients included in the cohort are given in supplementary table 1. Our clinical sequencing cohort of 34,036 patients with cancer of which 60% are biopsied from the primary lesion. 98% of our cohort comprised of adult patients. Age of patients at clinical sequencing ranged from 18 to 96 years. Female patients comprised 55% of the cohort.
Recruitment	The study cohort comprised of 34,036 patients who underwent prospective clinical sequencing as part of their care at Memorial Sloan Kettering Cancer between February 2014 and July 2019, representing all solid tumors. All patients for whom both tumor and normal specimens were sequenced are included in the present study.
Ethics oversight	This study was approved by the Memorial Sloan Kettering Cancer Center Institutional Review Board, and all patients provided written informed consent for tumor sequencing and review of medical records for demographic, clinical and pathology information.

Note that full information on the approval of the study protocol must also be provided in the manuscript.

Clinical data

Policy information about clinical studies

All manuscripts should comply with the ICMJEguidelines for publication of clinical research and a completedCONSORT checklist must be included with all submissions.

Clinical trial registration	NCT01775072

Study protocol	The protocol for prospective tumor sequencing of advanced cancers is available at clinicaltrials.gov, NCT01775072. Our study represents a retrospective anlaysis of clinical sequence data generated through that study, which was approved as a retrospective research protocol by the MSK institutional review board.
Data collection	The study cohort was comprised of 34,036 patients. All patients underwent prospective clinical sequencing as part of their clinical care (February 2014 to July 2019) in the CLIA-compliant Molecular Diagnostics Service at MSKCC .
Outcomes	No outcomes were reported.
